# Effects of Microplastics on Immune Responses of the Yellow Catfish *Pelteobagrus fulvidraco* Under Hypoxia

**DOI:** 10.3389/fphys.2021.753999

**Published:** 2021-09-21

**Authors:** Li'ang Li, Ran Xu, Lingfeng Jiang, Elvis Genbo Xu, Man Wang, Jie Wang, Bo Li, Menghong Hu, Lei Zhang, Youji Wang

**Affiliations:** ^1^International Research Center for Marine Biosciences at Shanghai Ocean University, Ministry of Science and Technology, Shanghai Ocean University, Shanghai, China; ^2^Huai'an Research Centre, Institute of Hydrobiology, Chinese Academy of Sciences, Huai'an, China; ^3^Key Laboratory of Exploration and Utilization of Aquatic Genetic Resources, Ministry of Education, Shanghai Ocean University, Shanghai, China; ^4^Department of Biology, University of Southern Denmark, Odense, Denmark; ^5^The Key Laboratory of Aquatic Biodiversity and Conservation of Chinese Academy of Sciences, Institute of Hydrobiology, Chinese Academy of Sciences, Wuhan, China; ^6^University of Chinese Academy of Sciences, Beijing, China; ^7^Fisheries Research Institute, Wuhan Academy of Agricultural Sciences, Wuhan, China

**Keywords:** microplastics, hypoxia, *Pelteobagrus fulvidraco*, immune response, specific growth rate

## Abstract

Compared with marine organisms, research on microplastics (MPs) in freshwater organisms is still less although MPs have been widely found in the freshwater ecosystem. Hypoxia is a ubiquitous issue in freshwater aquaculture, and under such scenarios, the toxic effects of MPs on typical aquaculture fish need to be clarified. In this study, we studied the effects of MPs (polystyrene) on specific growth rate (SGR), hypoxia-inducible factor-1α (HIF-1α), tumor necrosis factor-α (TNF-α), interleukin-8 (IL-8), and interferon (IFN) in the yellow catfish (*Pelteobagrus fulvidraco*) under hypoxic conditions. After 15 days of exposure, the SGR was not affected by MPs or hypoxia. MPs significantly increased the expressions of HIF-1α and TNF-α but inhibited the expression of IFN at high concentration MPs under normoxia. However, hypoxia significantly inhibited the expression of IL-8 and TNF-α under high MP concentration and low MP concentration, respectively. In addition, MPs had significant concentration-dependent inhibitory effects on IFN under hypoxia. Surprisingly, a positive correction between HIF-1α and TNF-α was found in fish. Although hypoxia might alleviate the effects of MPs with low concentrations, the interaction of hypoxia and MPs aggravated the negative effects of MPs on immune factors at high concentration MPs. This study provided new insight into the complex effects of hypoxia and MPs on aquatic organisms, and future studies should focus on the cellular pathways of immune cells in fish. Given that MPs could induce the immune response in fish, considerations should be paid to the impacts of MPs on freshwater aquaculture, and hypoxia should be taken into consideration when evaluating the effects of MPs.

## Introduction

Microplastics (MPs, <5 mm in size) mainly come from the daily life of people or from the aging, weathering, and broken of the large plastic pieces (Cole et al., 2011). Due to their small volume, MPs are difficult to remove by sewage treatment completely (Hamidian et al., 2021), which make them able to flow into inland rivers and eventually into marine ecosystems (Wang et al., 2021a). In recent years, many studies have been made on the occurrence of MPs in the environment and their effects on marine organisms, including physical damage, behavior change, tissue lesions, oxidative stress, and gene damage (Huang W. et al., 2021). MPs can also be used as a carrier for some pollutants in the environment and carry the pollutants into the body when organisms ingest MPs, which may cause more serious damages to organisms (Gu et al., 2020).

With the increasing discoveries of MPs in rivers and lakes, MPs have been increasingly viewed as a serious health concern to freshwater ecosystems (Shang et al., 2020). China is a major country in traditional aquaculture (Su et al., 2020), and freshwater aquaculture products account for more than half of the total production in 2020 (Fisheries Administration Ministry of Agriculture Rural Affairs, 2021). In recent years, MPs have been found in major rivers and lakes in China such as Yangtze River, Yellow River, Pearl River, Poyang Lake, Dongting Lake, and Taihu Lake (Fu and Wang, 2019). Especially in Taihu Lake, the concentration of MPs has reached an astonishing 25,800 items/m^3^ (Su et al., 2016), which even greatly exceeds the Yellow Sea (0.33 items/m^3^) and South China Sea (2,569 items/m^3^) (Cai et al., 2018; Wang et al., 2018). In addition to the contamination of MPs in freshwater aquaculture water sources, various freshwater aquaculture patterns, such as cycled-water, cement-pool, net-cage, rice-field, and fish-light, can release MPs into aquaculture waters (Sahu et al., 2016; Lv et al., 2020). Due to the multiple sources of MPs, freshwater aquaculture objects are inevitably subjected to MP pollution. However, less is known on the effects of MPs on freshwater species, especially economic freshwater fish.

In recent years, the harm of MPs to the immune system of fish by affecting the expression of immune genes in fish has been revealed. Tumor necrosis factor-α (TNF-α), interleukin (IL), and interferon (IFN) are important factors in the immune system, which play the role of activating immune cells, regulating immune cells, and activating antiviral cells, respectively (Samuel, 2001; Whyte, 2007). Huang et al. (2020b) showed that the expressions of TNF-α, IL-6, and IFN in the liver of the guppy *Poecilia reticulata* were significantly increased after 28 days of exposure to MPs. What is more, MPs not only affect the expression of IFN at the gene level but also increase the secretion of the protein corresponding to IFN (Jin et al., 2018). Luo et al. (2021) found that MPs induced the expression of increased IL-8 compared with the control group. The expression changes of these proinflammatory biomarkers under the MPs make them become the important index on the evaluation of fish welfare.

Hypoxia, a common phenomenon in aquaculture, which can be caused by temperature rise, eutrophication, high-density aquaculture, and water pollution (Wang M. et al., 2021), often occurs at the bottom of the water body, leading to behavioral change (Xu et al., 2006), metabolic obstruction (Obirikorang et al., 2020), and decreased immunity (Ngoepe et al., 2020) of aquaculture objects. What is more, hypoxia can aggravate the negative effects of other environmental stressors on aquatic animals (Hu et al., 2016; Somo et al., 2020), thus reducing the production efficiency of aquaculture. With the deepening of research, the hypoxia-inducible factor (HIF) is found to be the main transcription factor regulating the hypoxia signaling pathway in vertebrates (Wang C. et al., 2020). HIF-1 is widely used in the study of hypoxia (Xu et al., 2021), and the α subunit of HIF-1 is most sensitive to hypoxia compared with the β subunit (Abdel-Tawwab et al., 2019). Although this subunit has a short half-life in cells, it is difficult to be hydrolyzed under hypoxia and accumulates in large quantities in cells, which can activate the hypoxia signaling pathway (He et al., 2019). At present, the HIF-1α gene sequence has been successfully cloned from an aquaculture object, and its function in the hypoxia signaling pathway has been found (Lin et al., 2021). In addition, other studies have shown that HIF-1α plays an important role in regulating inflammation, but this effect has only been found in higher vertebrates such as human cells and mouse cells (Li et al., 2020; Pena et al., 2020).

On the one hand, MPs are newly emerging environmental pollutants in freshwater aquaculture, which have been confirmed the biological accumulation of aquaculture species (Lv et al., 2020). On the other hand, hypoxia is a traditional inhibitor of aquaculture and often occurs in an aquatic pond (Xu et al., 2006). However, the effect of these two simultaneous factors at the bottom of the pond on benthic objects is unknown.

The yellow catfish *Pelteobagrus fulvidraco* is a unique freshwater aquaculture species in China, which is widely loved for its delicious meat. Its production reached 565,477 tons in 2020 (Fisheries Administration, Ministry of Agriculture and Rural Affairs, People's Republic of China, 2021). Hypoxia occurs frequently in yellow catfish aquaculture due to the intensive, high-density, and high-feeding farming, while MPs were also detected from yellow catfish in a reservoir (Zhang et al., 2017; Wang M. et al., 2021). However, this research on yellow catfish mainly focuses on breeding and nutrition (Wang et al., 2021b; Zhao et al., 2021), while the information on the complex effects of MPs and hypoxia on its physiology and ecology is very scarce. In contrast, compared with other freshwater aquaculture fish, their scaleless bodies make them more vulnerable to MPs and their immune function more important (Feng et al., 2019), and the demersal habits of yellow catfish make them more susceptible to MPs and hypoxia. Therefore, we aimed to study the specific growth rate (SGR) and immune-associated genes, i.e., HIF-1α, TNF-α, IL-8, and IFN of yellow catfish under combined exposure of hypoxia and MPs. We concluded that (1) MPs cause the cellular immunological stress of yellow catfish and (2) hypoxia aggravates the negative effects of MPs on immune parameters of yellow catfish at a high concentration.

## Materials and Methods

### Ethics Statement

The processes involving animals complied with the Animal Research: Reporting of *in vivo* Experiments (ARRIVE) guidelines. All experiments were conducted under the approval of the research committee of the Institute of Hydrobiology, Chinese Academy of Sciences.

### Fish

The juvenile yellow catfish (size 5.80 ± 0.31 cm, body weight 3.00 ± 0.74 g) were collected from Huai'an Research Center of Hydrobiology Research Institute, Chinese Academy of Sciences. Before the experiment, 1,000 yellow catfish were acclimated for 2 weeks in indoor tanks with a circulating water filtration system. During the domestication process, water quality parameters were controlled as follows: temperature 27.2 ± 0.4°C, pH 7.1 ± 0.62, dissolved oxygen (DO) 6.73 ± 0.3 mg/L, and ammonia nitrogen <0.1 mg/L. All these parameters were measured by using YSI Professional Plus (Ohio, USA). The fish were fed an expanded pellet diet (1.8 g/day) containing more than 40% of protein at 08:00 and 18:00 (Qianjiang Jiajia Biotechnology Co., Ltd., Qianjiang, China) every day.

### Microplastics and Hypoxia

The MPs (polystyrene, green fluorescent microsphere, 20 μm, density: 10 mg/cm^3^, and excitation and emission peaks: 488 and 518 nm) used in the experiment were purchased from Tianjin Baseline Chromtech Research Centre, Tianjin, China. Based on Wang X. et al. (2020), before the exposure experiment, a scanning electron microscope (SEM, S-3400N, Hitachi, Japan) and a micro-Fourier transform infrared spectroscope (m-FT-IR, NICOLET iN10, Thermo Fisher Scientific, USA) were used to detect and validate the MPs used in the experiment, respectively. According to the study by Fu and Wang (2019), in the Chinese freshwater ecosystem, the most serious polluted level and the main size of MPs are 25,800 particles/m^3^ and 20 μm, respectively. Thus, two MP concentrations, i.e., 25.8 particles/L (about 0.115 μg/L, represented the environmental concentration) and 2,580 particles/L (about 11.5 μg/L, represented the high concentration), as well as the control treatment (0 particles/L) were set for the experiment. A stock solution of MPs in ultrapure water was prepared and then sonicated 30 min before using for the experiment (Li et al., 2021).

Based on the DO in acclimation and the study by Diaz and Rosenberg (2008), the normoxia and hypoxia were set to 6.7 and 2.0 mg/L, respectively. The hypoxic condition in the experimental tank was achieved through the fish respiration consumption and the adjustment of the air stone in the tank. This method has been verified in the experiment by Wang M. et al. (2021), and the DO can be reduced to 2.0 mg/L within 2 h. In short, before the beginning of hypoxic exposure, the tank was sealed with kraft paper, and the aeration device was closed. The kraft paper was opened when the DO reached the specified value, which was detected by YSI Professional Plus, and the aeration amount of the aeration device was controlled to stabilize the DO.

### Exposure Experiment

The exposure lasted for 15 days and included six groups, namely, normoxia + no-MPs, normoxia + low concentration MPs, normoxia + high concentration MPs, hypoxia + no-MPs, hypoxia + low concentration MPs, and hypoxia + high concentration MPs. After the acclimation, a total of 540 healthy fish was randomly sampled and divided averagely into six experimental groups, and each group had three repeated tanks (560 × 350 × 340 mm, with 30 L of water, *N* = 30).

At the beginning of the experiment, MPs were added to the relevant tanks to achieve the desired concentrations. To maintain water quality and concentrations of MPs, water in each tank was renewed for 1 s every day (appropriate modifications were made on the basis of Huang et al., 2020a), and then the measures mentioned above were repeated to make sure each tank meet the exposure condition. Except for DO, all water parameters during the experiment were similar to the acclimation. In addition, the survival of the fish was recorded after the renewal of the water.

### Sampling

After 15 days of exposure, the weight of fish from each tank was recorded (*N* = 4). Then the gill (the major organ for breath) and liver (the major organ for detoxification) were collected from them which were anesthetized by 120 mg/L of 3-aminobenzoic acid ethyl ester methanesulfonate (MS-222, Cat. No. A5040, Sigma-Aldrich., Shanghai, China) and put into the liquid nitrogen to freeze quickly, and the gills and livers of four fish in each tank were mixed into one sample. All the samples were stocked in −80°C refrigerator for biochemical analysis.

### Quantitative PCR

The method of Trizol was used to extract the total RNA from the gills and livers, and the concentration and purity of RNA were determined by using a microspectrophotometer (Thermo Fisher Scientific, USA). The cDNA was synthesized with total RNA using the reverse transcription kit [PrimeScript (*r*) RT Reagent Kit with gDNA Eraser] according to the instructions.

After determining the amplification efficiency of primers (>90%, Table 1), the relative expressions of HIF-1α (in gills), TNF, IL-8, and IFN (in livers) were detected by qPCR. The qPCR was conducted on the 7500 Real-Time PCR System (Applied Biosystems, USA) using the 2× SYBR Green qPCR Mix (Antibody, ROX) (Genenode Biotech Ltd., Cat# 4302). The mixture of qPCR included 1 μl of cDNA, 0.25 μl of forward and reverse primers, and 5 μl of 2× SYBR Green qPCR Mix (Antibody), and the RNase-free ddH_2_O were added to fill a total volume of 10 μl. After full mixing, the following qPCR procedures were executed: 95°C for 3 min, followed by 40 cycles of 95°C for 15 s and 60°C for 30 s. Finally, the relative expression levels were measured by using the 2^−ΔΔCt^ method using β-actin as an internal reference.

**Table 1:** Primers for qPCR identification of HIF-1α, TNF, IL-8, IFN, and β-actin.

**Gene**		**Primer sequence (5^**′**^-3^**′**^)**
HIF-1α	F	CGGATCCAGAGCAAAGCGAT
	R	TTAGCATGACGTCGTCTCCG
TNF	F	ATAACCCACGCCTATGACTG
	R	GGCTATGACTCGCAACACTT
IL-8	F	CACTCACCAAGCCAGCAATG
	R	AGACAACCCAAGACTTCACC
IFN	F	AGAGGCAAGGAGTCTGAGGTATT
	R	CCAGGTGAGAGGTGACATTGTG
β-actin	F	TTCGCTGGAGATGATGCT
	R	CGTGCTCAATGGGGTACT

### Statistical Analysis

The SGR was calculated by the following formula according to the study by Yang et al. (2015) by using Excel 2019:

Specific growth rate (SGR, %) = 100 × [ln (*W*_t_) – ln(*W*_0_)]/*t* where *W*_t_ (g) and *W*_0_ (g) are the final and initial fish body weights, respectively, and *t* means duration.

The results were expressed as mean ± SD. After verifying the distribution normality of the data by using the Kolmogorov–Smirnov test, two-way ANOVA was used to analyze the effects of MPs, hypoxia, and their interaction. Then, the effects of MPs at each fixed DO and the effects of DO at each MP concentration were evaluated by using the one-way ANOVA and Student's *t*-test, respectively. Significance was indicated by *P* < 0.05. For all biochemical parameters, the principal component analysis (PCA) was used to reduce the complexity of multiple index analyses. The Pearson coefficient (*r*) was used to compute the correlation between variables, and only the correlations with | *r* | > 0.4 and *P* < 0.05 were considered to perform the unary line fitting. All the analysis and data visualizations (including SGR) are conducted on the Origin Pro 2018C.

## Results

### Characteristics of MPs

**Figure 1:**
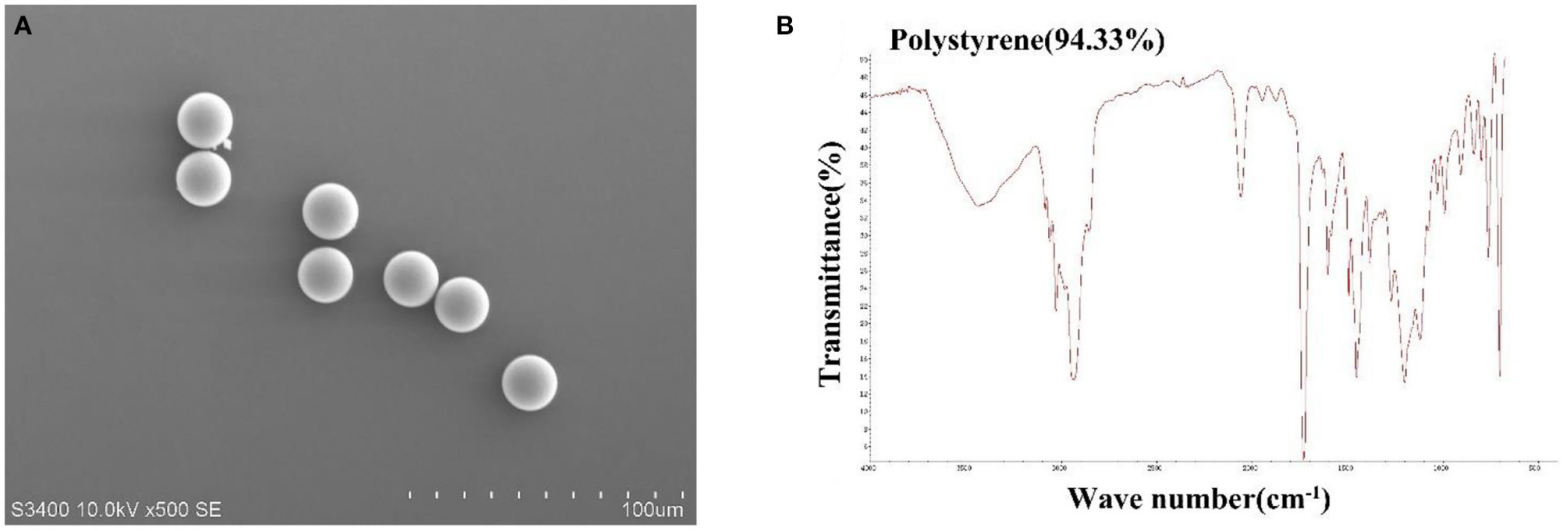
Scanning electron microscope (SEM) image of MPs **(A)** and micro-Fourier transform infrared (m-FT-IR) spectroscopy of MPs **(B)**.

The spectrum match of MPs according to m-FT-IR was 94% (Figure 1A). The shape of MPs is smooth sphere, and the particle size is 20.2 ± 0.04 μm (*N* = 5) according to SEM (Figure 1B).

### Dissolved Oxygen

**Figure 2:**
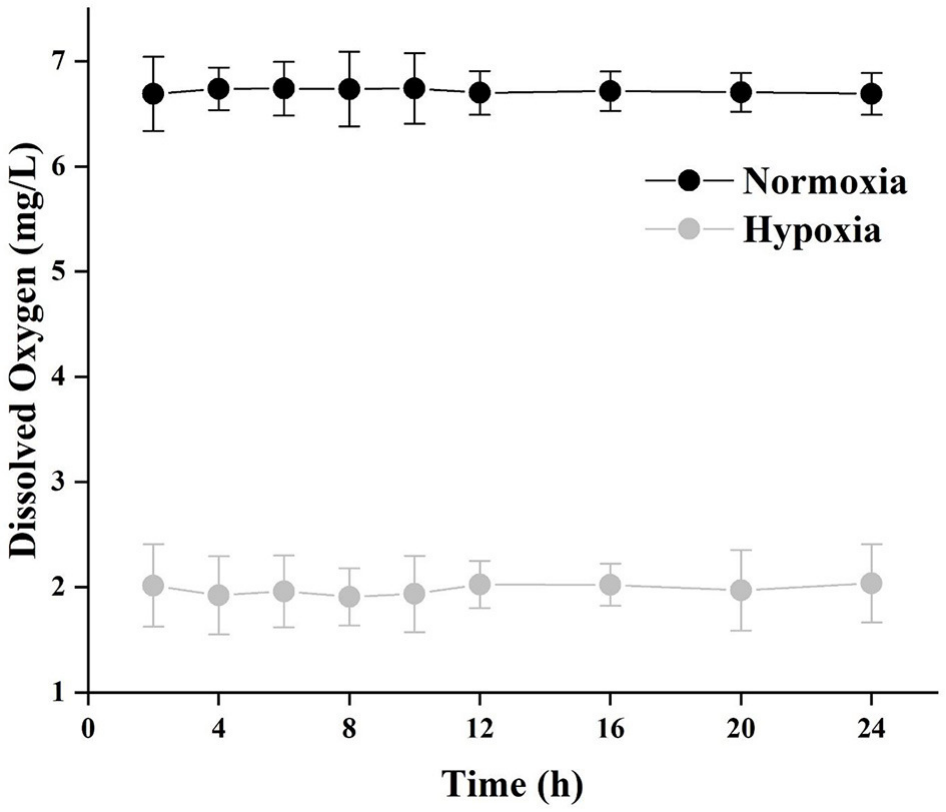
The data of dissolved oxygen (DO) under normoxia and hypoxia after renewing the water every day (*N* = 15). The *X*-axis is the time after renewing the water, and the *Y*-axis is the DO at the corresponding time.

The DO for normoxia and hypoxia treatments every day is shown in Figure 2 (*N* = 15). Two hours after renewing the water, the DO of hypoxia groups reached 2.02 ± 0.39 mg/L and was maintained at this level until the next water renewing. The DO of normoxia groups was 6.72 ± 0.25 mg/L.

### Specific Growth Rate

**Figure 3:**
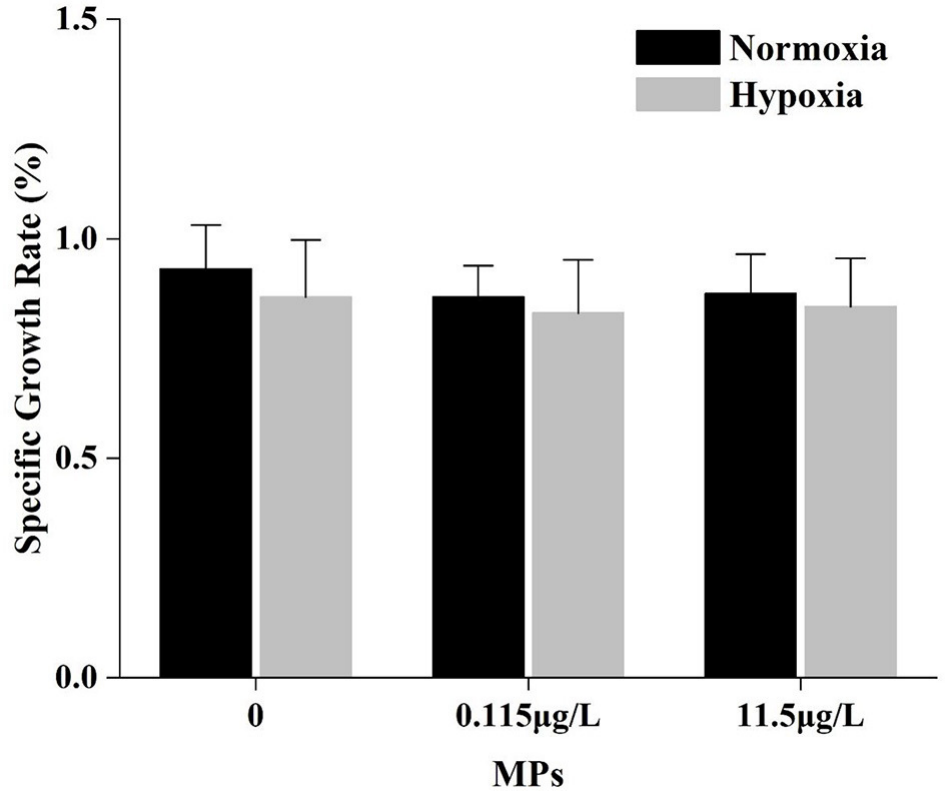
The specific growth rate (%) of *Pelteobagrus fulvidraco* after exposure of 15 days.

No significance was observed in the interaction between MPs and DO in SGR (Table 2). Although the SGRs in hypoxia are all lower than in normoxia regardless of MP concentrations, there was no statistical difference between treatments (*P* > 0.05, Figure 3).

**Table 2:** Summary of two-way ANOVA results on effects of microplastics (MPs) and dissolved oxygen (DO) on SGR, HIF-1α, TNF-α, IL-8, and IFN of *Pelteobagrus fulvidraco*.

**Source**	**df**	**HIF-1α**			**TNF-α**			**IL-8**		
		**MS**	***F***	***P***	**MS**	***F***	***P***	**MS**	***F***	***P***
MPs	2	1.513	2.326	0.140	0.655	34.926	<0.01	0.341	8.536	<0.01
DO	1	0.027	0.041	0.842	<0.01	0.031	0.863	0.080	2.009	0.182
MPs × DO	2	0.617	0.948	0.415	0.191	10.182	<0.01	0.282	7.057	<0.01
Error	12	0.650			0.019			0.040		
**Source**	**df**	**IFN**			**SGR**					
		**MS**	**F**	**P**	**MS**	**F**	**P**			
MPs	2	1.047	131.158	<0.01	<0.01	0.383	0.690			
DO	1	0.084	10.500	0.007	0.008	0.737	0.407			
MPs × DO	2	0.599	75.098	<0.01	<0.01	0.04	0.961			
Error	12	0.008			0.011					

### The Relative Expression of HIF-1α

**Figure 4:**
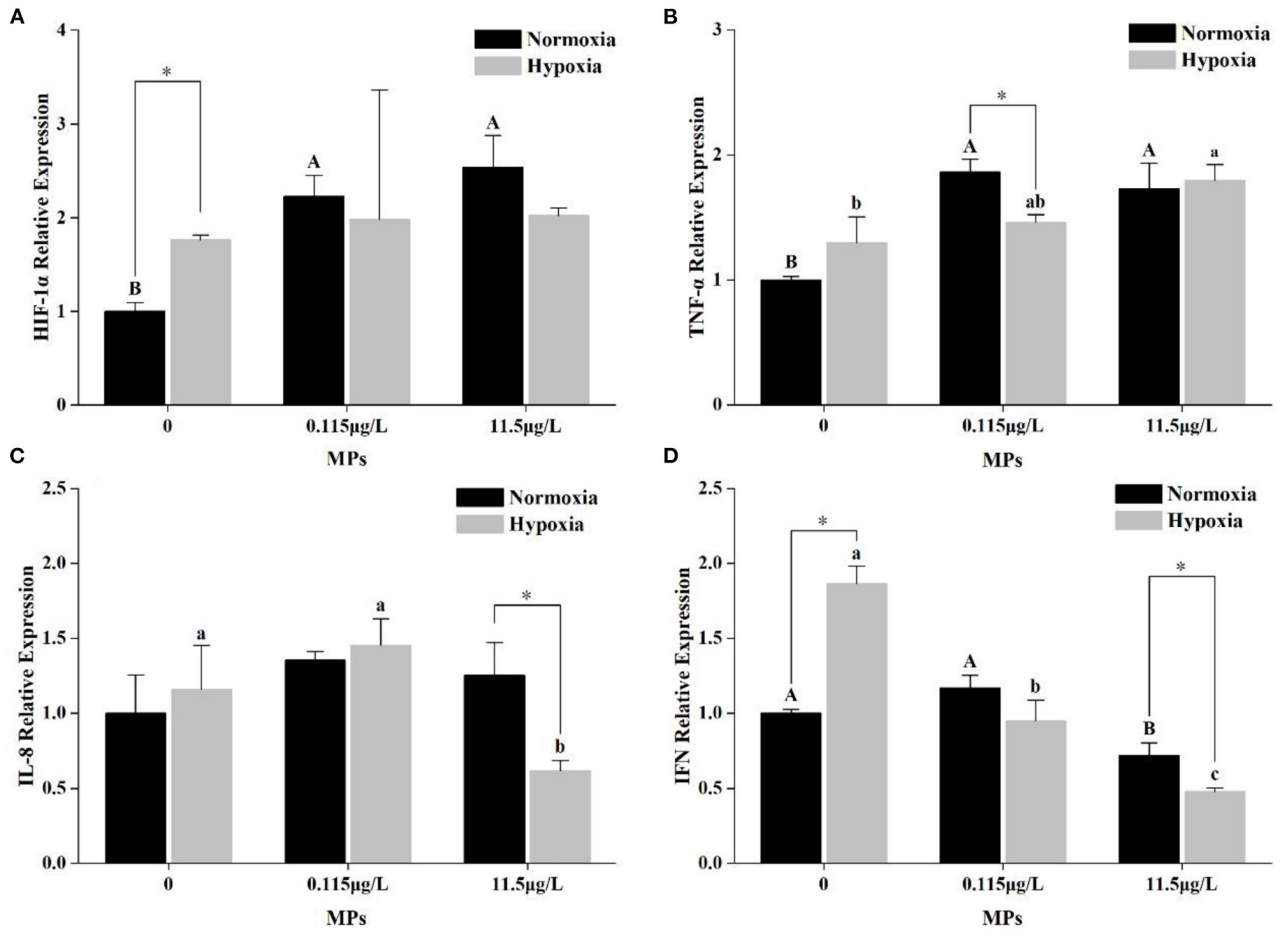
Relative expression of HIF-1α **(A)**, TNF **(B)**, IL-8 **(C)**, and IFN **(D)** under different treatments (normoxia + 0 MPs, normoxia + 0.115 μg/L MPs, normoxia + 11.5 μg/L MPs, hypoxia + 0 MPs, hypoxia + 0.115 μg/L MPs, and hypoxia + 11.5 μg/L MPs) of *P. fulvidraco*. Different capital letters and different lowercase letters indicate significant differences among MP concentrations in normoxia and hypoxia (*P* < 0.05). “*” represents the significant difference between normoxia and hypoxia.

There was no significant interactive effect of MPs and DO on HIF-1α (Table 2). After 15 days of exposure, HIF-1α was increased in both 0.115 and 11.5 μg/L MPs significantly compared with the control (0 μg/L) under normoxia (*P* < 0.05). However, under hypoxia, no significant difference among the three MP treatments was observed. Compared with the normoxia, hypoxia significantly increased HIF-1α when there were no MPs but significantly reduced HIF-1α under high MP treatment (11.5 μg/L) (*P* < 0.05, Figure 4A).

### The Relative Expression of TNF-α, IL-8, and IFN

There were significant interactions between MPs and DO on TNF-α, IL-8, and IFN (Table 2).

In normoxia, TNF-α was significantly increased in 0.115 and 11.5 μg/L MPs compared with the control (0 μg/L) (Figure 4B, *P* < 0.05). There was no significant difference in IL-8 among three MP treatments under normoxia (Figure 4C, *P* < 0.05). However, compared with the 0 and 0.115 μg/L MPs, IFN was decreased in 11.5 μg/L MPs under normoxia (Figure 4D, *P* < 0.05). Under hypoxia, TNF and IL-8 were significantly increased and significantly decreased in 11.5 μg/L MPs compared with the control, respectively (Figures 4B,C, *P* < 0.05). Surprisingly, there was a significant concentration-dependent decrease in IFN in hypoxia (Figure 4D, *P* < 0.05).

In non-MP treatment, IFN in hypoxia was higher than those in normoxia significantly (Figure 4D, *P* < 0.05). Only TNF in hypoxia was lower than that in normoxia under 0.115 μg/L MP treatment (Figure 4B, *P* < 0.05). In addition, there were significantly lower expressions of IL-8 and IFN under hypoxia than normoxia in 11.5 μg/L MPs (Figures 4C,D, *P* < 0.05).

### PCA and Correlation Analysis

**Figure 5:**
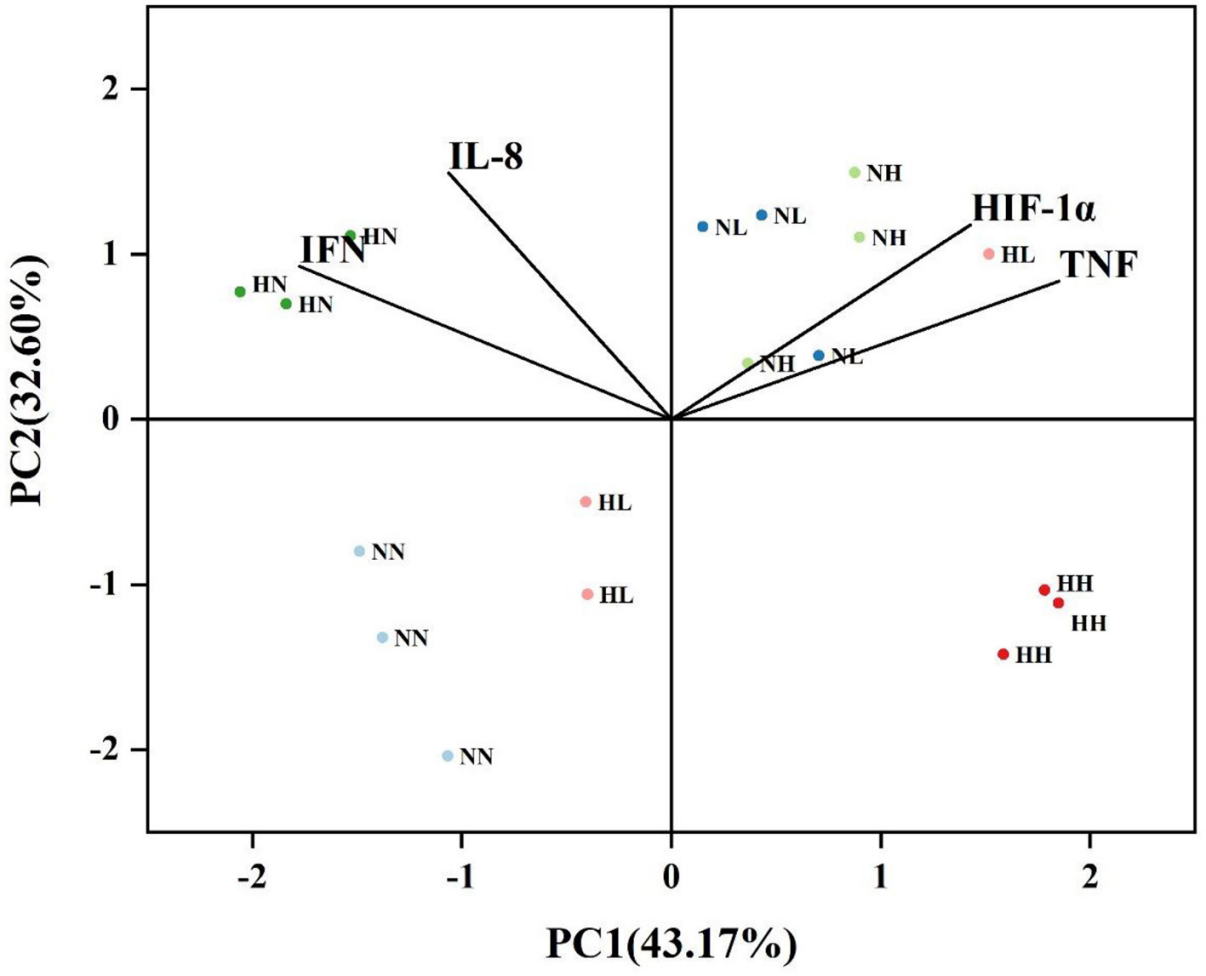
The PCA of *P. fulvidraco* for 15 days of exposure (NN: normoxia + 0 MPs, NL: normoxia + 0.115 μg/L MPs, NH: normoxia + 11.5 μg/L MPs, HN: hypoxia + 0 MPs, HL: hypoxia + 0.115 μg/L MPs, and HH: hypoxia + 11.5 μg/L MPs).

As shown by PCA, PC1 accounted for 43.17% of all variables, which separated the presence or absence of MP treatments. PC2 accounted for 32.60% of all variables, which separated normoxia and hypoxia (Figure 5).

The Pearson coefficient (*r*) between variables is shown in Table 3, and the *r* between TNF and HIF-1α and IL-8 and IFN was greater than 0.4. However, only the *r* between TNF and HIF-1α was considered according to the standard mentioned above (*r* = 0.49129 > 0.4, *P* = 0.0384 < 0.05). According to the scatter diagram, we chose the unary equation, the sine function, and the logarithmic function to fit the data, compared the fitting degree of the three models, and determined that the unary equation was the most suitable model (Supplementary Table S1).

**Table 3:** The Pearson coefficient (*r*) between HIF-1α, TNF, IL-8, and IFN of *P. fulvidraco*.

		**HIF-1α**	**TNF**	**IL-8**	**IFN**
HIF-1α	Pearson coefficient (*r*)	1	0.49129	0.01644	−0.08288
	*P* value	–	0.0384	0.94839	0.74371
TNF	Pearson coefficient (*r*)	0.49129	1	0.01041	−0.36829
	*P* value	0.0384	–	0.96729	0.13264
IL-8	Pearson coefficient (*r*)	0.01644	0.01041	1	0.45988
	*P* value	0.94839	0.96729	–	0.05483
IFN	Pearson coefficient (*r*)	−0.08288	−0.36829	0.45988	1
	*P* value	0.74371	0.13264	0.05483	–

**Figure 6:**
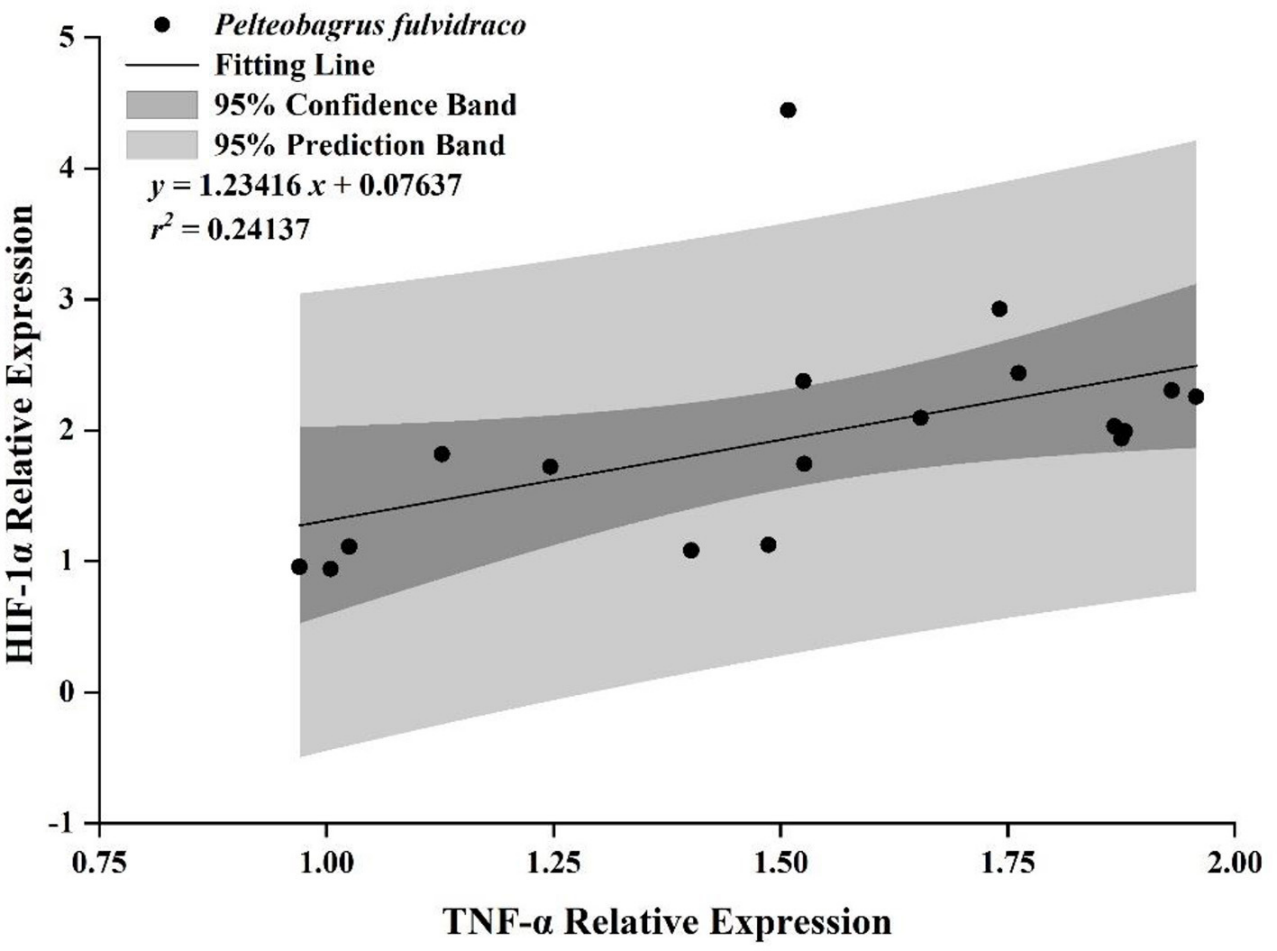
The correlation between the relative expression of HIF-1α and TNF of *P. fulvidraco* by unary linear regression.

Unary linear regression was the best model to fit the correlation between TNF and HIF-1α. The fitting equation was ***y* =** **1.23416*x* +** **0.07637 (*r***^**2**^ **=** **0.24137)** and all the data points was in 95% prediction bands except one (Figure 6).

## Discussion

Microplastics have become a hot topic since Thompson et al. (2004) discovered them. But there is still less research on MPs in freshwater organisms than in marine organisms. The Qinghai-Tibet Plateau is the birthplace of many rivers (e.g., the Yangtze River, the Yellow River, and the Lancang River). However, Jiang et al. (2019) found that the water bodies of the Qinghai-Tibet Plateau contained MPs, and the Yangtze River would receive more than 3.1 × 10^5^ tons MPs each year (Lebreton et al., 2017). Some studies have carried out the toxicological effects of MPs on tilapia (*Oreochromis niloticus*), but the size of MP particles (0.1 and 5 μm, respectively) and the exposure concentration (100 and 50 μg/L, respectively) are far from the results of the environmental investigation (Ding et al., 2018; Huang Y. et al., 2021). Based on Fu and Wang (2019), in Chinese freshwater ecosystem, the highest MP concentration is 25,800 items/m^3^, and the smallest size of MPs is just about 20 μm. Therefore, this study can provide environmentally relevant insights on MPs in the field of freshwater based on the real size and concentration in the environment.

The SGR was an important index to measure the economic benefits of fisheries, and the larger the value was, the greater of fish would be. Malinich et al. (2018) found that MPs had no impact on the growth of the fathead minnow *Pimephales promelas*, and a similar result was detected from the brown trout *Salmo trutta* (Jakubowska et al., 2020). However, MPs could induce growth inhibition in the common carp *Cyprinus carpio*. The reason for the difference may be the different exposure methods (waterborne or foodborne). In this study, yellow catfish did not exhibit a significant difference between MP treatments, and it might be associated with the smooth and spherical shape of MPs, which made them easily expel from the intestine (Mazurais et al., 2015). Hypoxia could inhibit the growth of juvenile fish, which had been reported previously (Yang et al., 2013; Campbell and Rice, 2014), and yellow catfish showed a significant decrease in SGR under hypoxia for 8 weeks (Yang et al., 2015). Under hypoxia, although there was no statistical difference, a minimal lower change could be seen compared with normoxia in only 15 days of exposure, which indicated that the hypoxia may impact the growth of yellow catfish regardless of the presence or absence of MPs.

Hypoxia can increase the expression of HIF-1α in organisms has been confirmed in a large number of studies (Kelly et al., 2020; Wang M. et al., 2021; Xu et al., 2021). HIF-1 is a heterodimer composed of HIF-1α and HIF-1β (Semenza, 1998). Among them, HIF-1β is insensitive to the changes in O_2_ availability, while HIF-1α can accumulate in large quantities *in vivo* due to its difficulty in degradation under hypoxia, activating the hypoxia signaling pathway (Uchida et al., 2004). Activated hypoxia signaling pathways modulate other biological processes associated with increased O_2_ release or decreased O_2_ consumption, such as the regulations of erythropoietin, glucose transporter, glycolytic enzyme, and expression of vascular endothelial growth factor (Semenza, 1999), allowing the organism to adapt to new environmental changes. This could also explain why the expression of HIF-1α changes significantly under hypoxia compared with normoxia. As shown in Figure 4A, both low (0.115 μg/L) and high (11.5 μg/L) concentration MPs could increase the expression of HIF-1α under normoxia. A similar result was obtained in the microcrustacean *Daphnia pulex* (Liu Z. et al., 2020), despite nanoplastics were used in their experiments, which might be more biotoxic than MPs (Browne et al., 2008). It was possible that HIF-1α could also be used as an indicator for toxicological tests of contaminants as well as other cytokines, such as the heat shock proteins (Varo et al., 2019; Jaikumar et al., 2021). Although this study did not detect the presence and quantity of MPs in gill tissues, it could be speculated from previous studies that the increased expression of HIF-1α might be related to the accumulation of MPs in gill tissues, which would influence the normal respiration (Lu et al., 2016; Wang et al., 2019; Huang et al., 2020a). However, in this study, we did not find that MPs increased the expression of HIF-1α under hypoxia treatment, indicating that MPs did not exert biological toxicity under hypoxia. As we know, fish will head out of the water to utilize the oxygen in the air when they live in the hypoxic water environment (Juca-Chagas, 2004), and this behavior is defined as “floating head.” During the experiment, in the hypoxia group, “floating head” was recorded. This behavior could decrease the amount of water fish filtered, reduce the number of MPs getting into fish, and thus ease the negative effects of MPs. Oxygen was necessary for the survival of oxygen-consuming organisms, and organisms had already developed an effective defense mechanism in the face of the occasional lack of oxygen during survival (Bickler and Buck, 2007). However, in the face of emerging environmental pollutants such as MPs, yellow catfish seemingly did not find an effective solution to resist the impacts of MPs, but hypoxia can hold back the effects of MPs on HIF-1α.

According to previous studies, harmful substances in the environment, such as MPs, heavy metals, pesticides, and antibiotics, could cause the inflammatory response of organisms (Liu H. et al., 2020; Maselli et al., 2020; Pirsaheb et al., 2020; Wang Y. et al., 2021). However, inflammatory responses in fish were often closely related to cytokines such as TNF, IL, and IFN (Falcão et al., 2021). TNF-α is an important immune factor that is heavily induced in injury, inflammation, and wound responses and activates macrophages, enhancing their phagocytosis and clearance to pathogens (Secombes et al., 2001). TNF-α has been shown to induce the expression of other genes involved in the immune response, such as IL-1β, IL-8, and COX2. IL-8, the first known chemokine, mainly attracts the movement of neutrophils, T-lymphocytes, and basophils in the body (Whyte, 2007). IFN is a secreted protein that induces antiviral activity in vertebrates and regulates apoptosis and cellular immunity (Samuel, 2001). Three factors interact to enhance the resistance of organisms to the external environment. Therefore, the transcription of these three types of immune factors was detected to comprehensively evaluate the immune status of yellow catfish.

In this study, the expressions of the three immune factors showed different changes under different MP concentrations. Under normoxia, the expressions of TNF-α were significantly increased with the presence of MPs. This suggested that MPs activated immune mechanisms in yellow catfish, which was due to the activation of the NF-κB signaling pathway, leading to the increased expression of related signaling factors (Wu et al., 2020; Yang et al., 2020). The same result was also obtained by Luo et al. (2021) who studied zebrafish (*Danio rerio*). Compared with normoxia, the expression of TNF-α was higher under hypoxia with 0 MPs, although this increase was not significant. Martinez et al. (2020) have found that hypoxia increased the expression level of TNF-α, and it was similar to our results. Under hypoxia, the relative expression of TNF-α was lower than those under normoxia in 0.115 μg/L MP treatment. But in 11.5 μg/L MP treatment, TNF-α was higher than those in 0.115 μg/L MPs under hypoxia. It is explained that hypoxia could reduce the negative effects of MPs on yellow catfish to some extent. The reasons for this result might be the reduced uptake of MPs by fish (direct reason) and the regulation of HIF-1α (indirect reason).

In contrast, the relative expression of IL-8 in normoxia was not influenced by the MPs, and it might be due to the size of MPs. Based on the experiment in *Danio rerio*, 0.5 μm MPs could decrease the relative expression of IL-8, but 50 μm MPs did not have the same negative effects (Jin et al., 2018), and the fact that smaller MPs have more serious negative effects has been proved by Browne et al. (2008). Under hypoxia, the variation tendency of IL-8 was the same as the normoxia condition which mainly showed an increase at 0.115 μg/L MPs and a decrease at 11.5 μg/L MPs, although the decrease at 11.5 μg/L was significant. In the study by Zhang et al. (2019) on the immune effects of di-2-ethylhexyl phthalate (DEHP, a kind of phthalic acid ester, PAEs) on yellow catfish *P. fulvidraco*, it was also observed that the relative expression of IL-8 increased at low concentration DEHP and decreased at high concentration DEHP. We held the opinion that this variation tendency of IL-8 was unique to yellow catfish exposed to environmental pollutants. However, the relative expression of IL-8 in 11.5 μg/L MPs in hypoxia was lower than that in normoxia, which explained that the composite effects of hypoxia and high concentration MPs could inhibit the expression of IL-8. And the interactive effects of hypoxia and MPs were observed in this study, which indicated that hypoxia aggravates the inhibition of hypoxia in the expression of IL-8. But this interaction did not have the same inhibition in 0.115 μg/L MPs under hypoxia, which might be related to the decrease of MP intake.

As a cytokine, IFN plays an important role in the immunity of the body, apoptosis, and antivirus (Samuel, 2001). Previous studies have shown that MPs can increase the relative expression of IFN in zebrafish (*Danio rerio*) and guppies (*Poecilia reticulata*) (Jin et al., 2018; Huang et al., 2020b). In this study, we also saw an increase in the relative expression of IFN in 0.115 μg/L MP treatment under normoxia, although this increase was not significant. It might be related to the inflammatory response caused by MPs. However, in the 11.5 μg/L MP group, the expression of IFN was significantly reduced under normoxia, which had not been found before. As mentioned above, it might be related to the difference of the species. Previous studies have shown that hypoxia could cause a significant increase in the expression of IFN (Niklasson et al., 2011; Chen et al., 2017), and the same result was obtained in this study. Based on the result of two-way ANOVA, a significant interaction of hypoxia and MPs was observed. This concentration-dependent interaction induced the inhibition in the expression of IFN.

According to the PCA, two principal components accounted for 75.77% of the total composition. PC1 separated MP treatment from non-MP treatment, accounting for 43.17 of the total variances, while PC2 separated normoxic treatment from hypoxic treatment, accounting for 32.60% of the total variances, indicating that MP treatment had a greater impact on yellow catfish. Significant positive correlation between TNF-α and HIF-1α, and this relationship had been discovered in mouse cells and human cells (van Uden et al., 2008; Li et al., 2020). We also found that there was a relationship between TNF-α and HIF-1α in fish, which could make up the gap between HIF-1α and TNF-α in fish. When inflammation occurred in the body, TNF-α induced a large number of macrophages to accumulate in the inflammatory site, consumed a large amount of oxygen, resulting in hypoxia in the inflammatory part and the production of a large number of HIF-1α (Cummins et al., 2016). In contrast, TNF-α could activate the NF-κB signaling pathway to produce NF-κB factor, and several subsequent NF-κB subunits bind to the promoter of HIF-1α, thereby inducing HIF-1α production in the body (Lin and Simon, 2016; Warbrick and Rabkin, 2019). In addition, HIF-1α plays a crucial role in the initiation, regulation, and coordination of cell responses during inflammation (Frede et al., 2007).

## Conclusion

This study filled the gap in understanding the effects of MPs on benthic fish under hypoxia. In this study, MPs were able to induce immune responses on yellow catfish. Hypoxia seemed to alleviate the effects of MPs at 0.115 μg/L on yellow catfish to some extent, but the interaction between hypoxia and MPs aggravated the negative effects of MPs on the expression of immune parameters on yellow catfish at 11.5 μg/L MPs. Thus, attention should be paid to the harm of MPs to aquaculture fish species, and the occurrence of hypoxia in water bodies should be reduced as far as possible. To form a systematic understanding, future studies should focus on the molecular pathway of immune cells in fish exposed to MPs and hypoxia.

## Data Availability Statement

The original contributions presented in the study are included in the article/Supplementary Material, further inquiries can be directed to the corresponding author/s.

## Ethics Statement

The animal study was reviewed and approved by Animal Research: Reporting of *In Vivo* Experiments (ARRIVE) guidelines Institute of Hydrobiology, Chinese Academy of Sciences.

## Author Contributions

LL: formal analysis, investigation, methodology, data curation, writing—original draft, and review and editing. RX: formal analysis, investigation, methodology, visualization, and writing—review and editing. LJ: investigation and methodology. EX and MH: investigation and writing—review and editing. MW, JW, and BL: investigation and methodology. LZ: funding acquisition, supervision, validation, and writing—review and editing. YW: conceptualization, supervision, validation, and writing—review and editing. All authors contributed to the article and approved the submitted version.

## Funding

This study was supported by the China Agriculture Research System of MOF and MARA (CARS-46) and the Subei Science and Technology Project of Jiangsu Province (Grant Nos.: SZ-HA2019017 and SZ-HAJH202005).

## Conflict of Interest

The authors declare that the research was conducted in the absence of any commercial or financial relationships that could be construed as a potential conflict of interest.

## Publisher's Note

All claims expressed in this article are solely those of the authors and do not necessarily represent those of their affiliated organizations, or those of the publisher, the editors and the reviewers. Any product that may be evaluated in this article, or claim that may be made by its manufacturer, is not guaranteed or endorsed by the publisher.
