# Supplementary material for: Effects of Microplastics on Immune Responses of the Yellow Catfish Pelteobagrus fulvidraco Under Hypoxia
Source: Front Physiol. 2021 Sep 21;12:753999. doi: 10.3389/fphys.2021.753999 (PMC8490880; doi:10.3389/fphys.2021.753999)
Supplement: Supplementary file 1 [file Data_Sheet_1.docx]

**Supplementary information for**

**Effects of microplastics on immune responses of the yellow catfish *Pelteobagrus fulvidraco* under hypoxia**

Li’ang Li ^a,c,e,#^, Ran Xu ^a,c,e,#^, Lingfeng Jiang ^a,e^, Elvis Genbo Xu ^g^, Man Wang ^b,c,d^, Jie Wang ^b,c,d^, Bo Li ^f^, Menghong Hu ^a,e^, Lei Zhang ^b,c,d,*^, Youji Wang ^a,e,*^

^a^ International Research Center for Marine Biosciences at Shanghai Ocean University, Ministry of Science and Technology, Shanghai Ocean University, Shanghai 201306, China

^b^ The Key Laboratory of Aquatic Biodiversity and Conservation of Chinese Academy of Sciences, Institute of Hydrobiology, Chinese Academy of Sciences, Wuhan 430072, China

^c^ Huai’an Research Centre, Institute of Hydrobiology, Chinese Academy of Sciences, Huai’an 223002, China

^d^ University of Chinese Academy of Sciences, Beijing 100049, China

^e^ Key Laboratory of Exploration and Utilization of Aquatic Genetic Resources, Ministry of Education, Shanghai Ocean University, Shanghai 201306, China

^f^ Fisheries Research Institute, Wuhan Academy of Agricultural Sciences, Wuhan 430207, China

^g^ Department of Biology, University of Southern Denmark, Odense M 5230, Denmark

^#^ These authors contributed equally to this work

**Figure S1.** The fitting results of TNF-α and HIF-1α by unary function.

**Figure S2.** The residuals of each fitting values of Y by unary function.

**Figure S3.** The fitting results of TNF-α and HIF-1α by sine function.

**Figure S4.** The residuals of each fitting values of Y by sine function.

**Figure S5.** The fitting results of TNF-α and HIF-1α by logarithmic function.

**Figure S6.** The residuals of each fitting values of Y by logarithmic function.


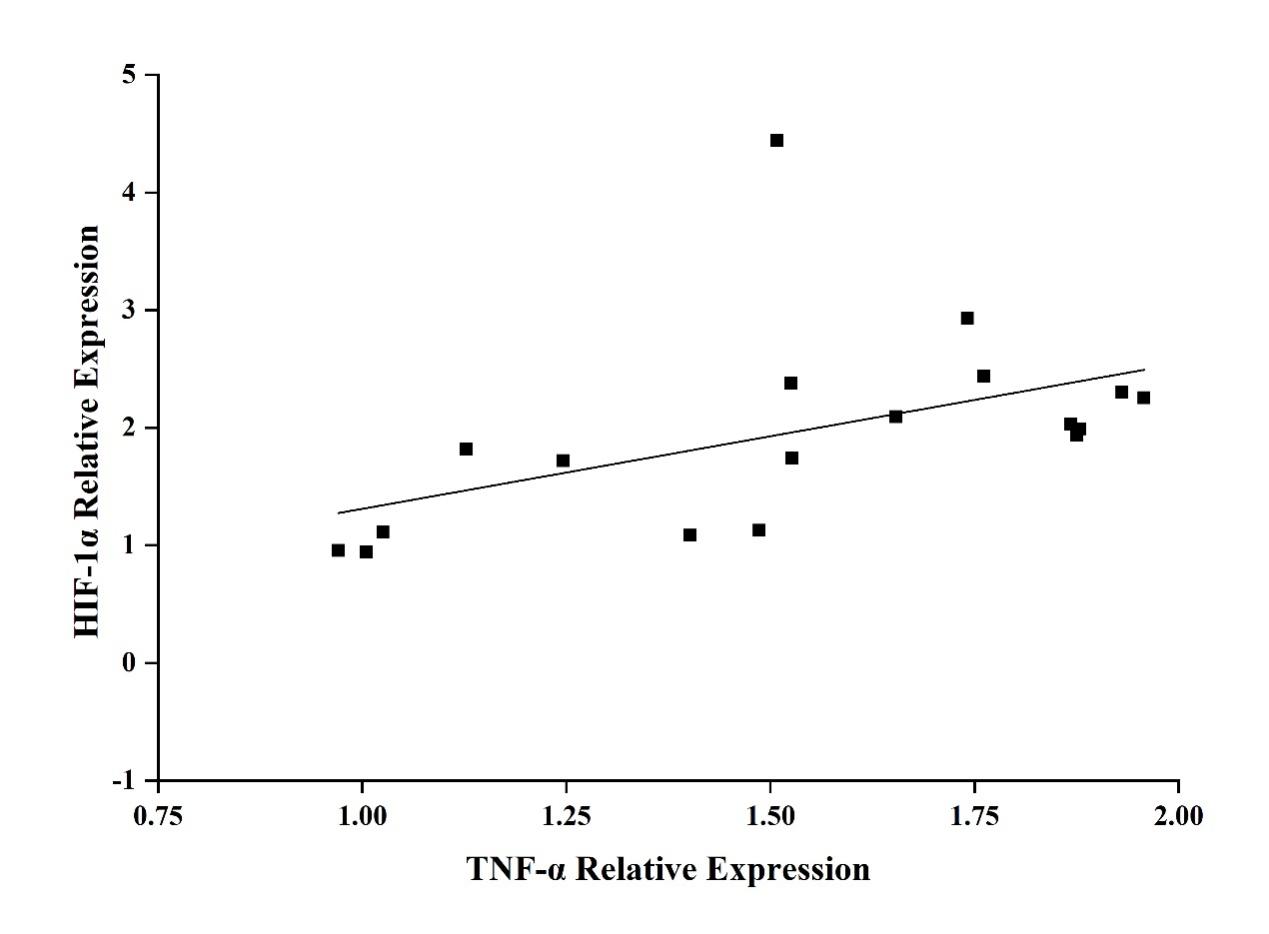


**Fig. S1**

**
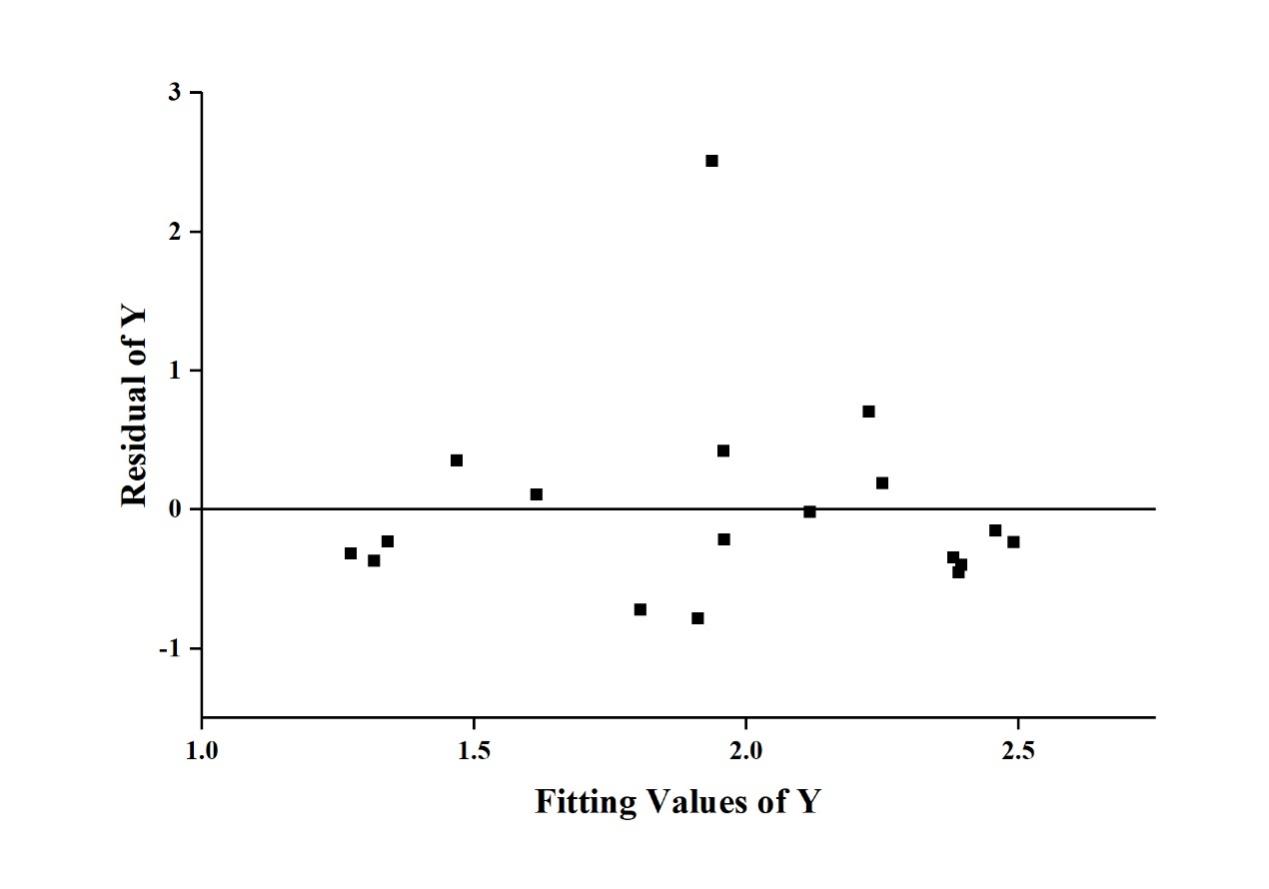
**

**Fig. S2**

**
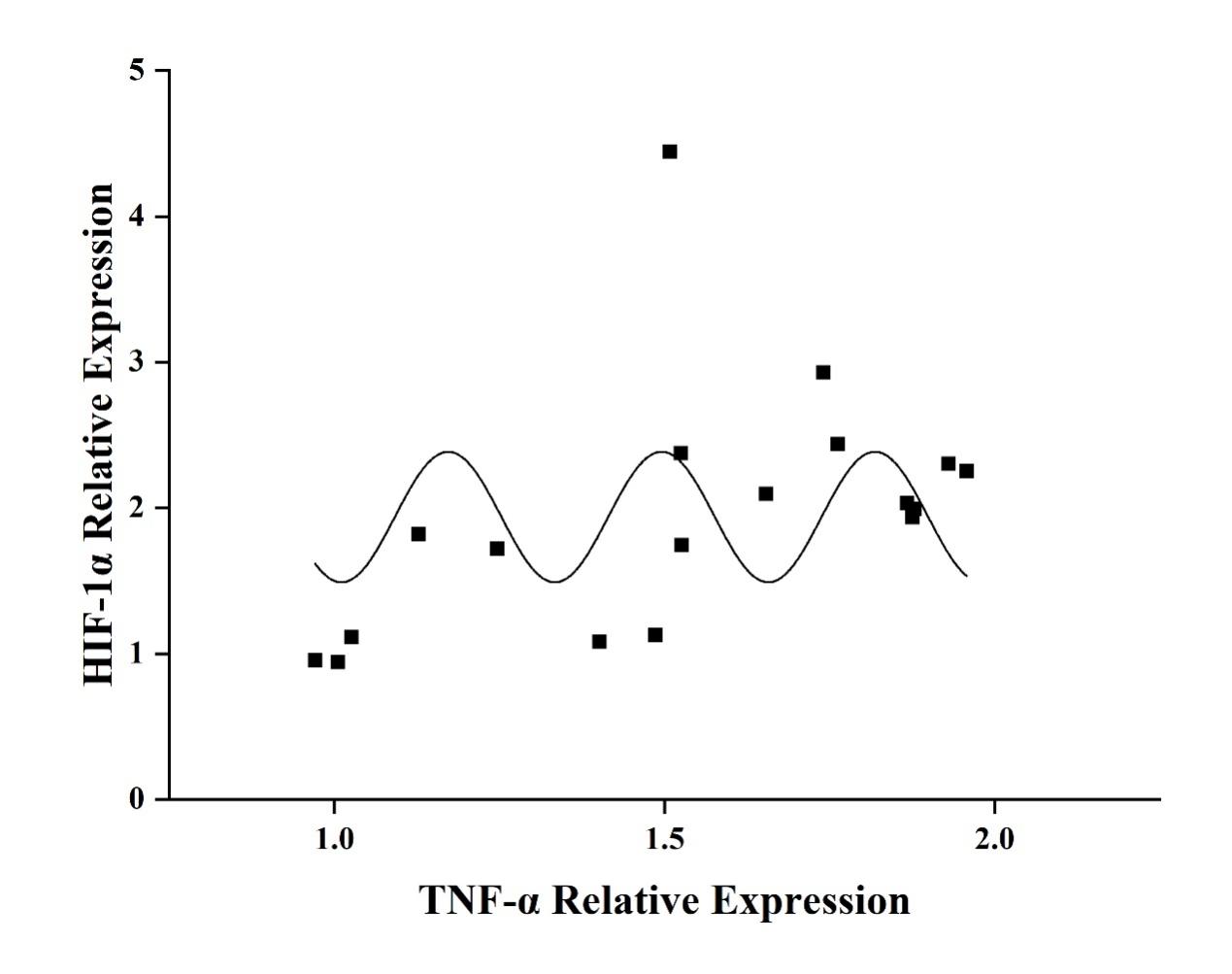
**

**Fig. S3**

**
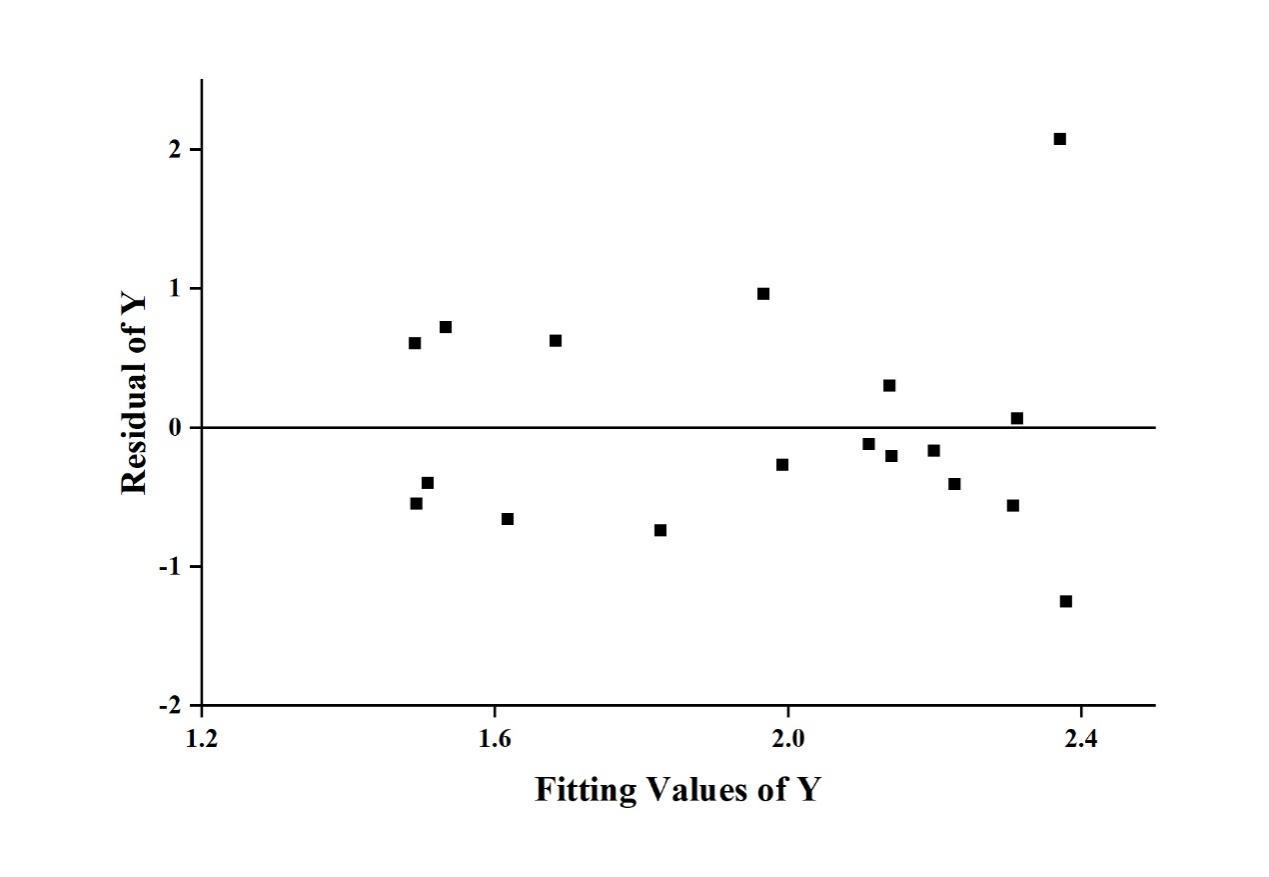
Fig. S4**

**
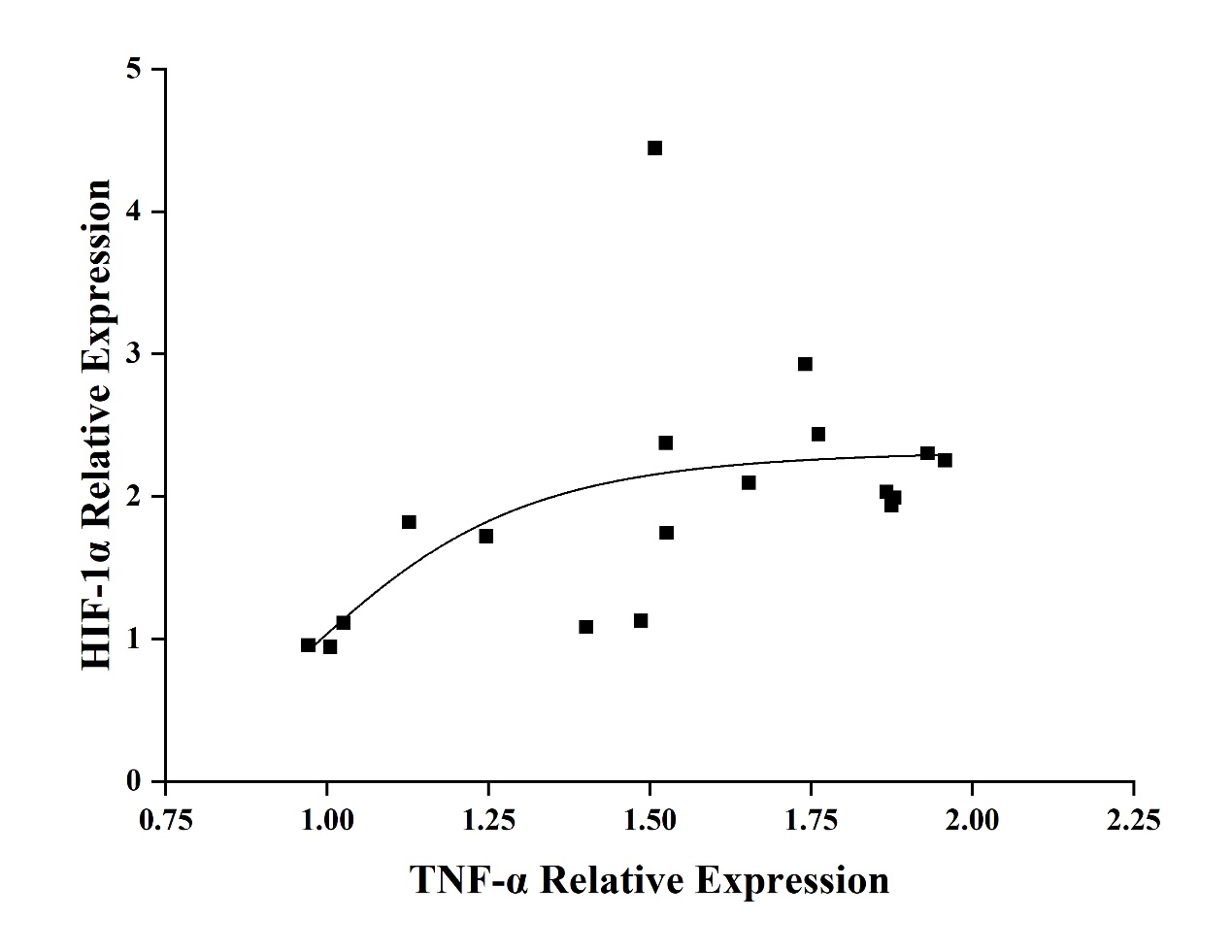
**

**Fig. S5**

**
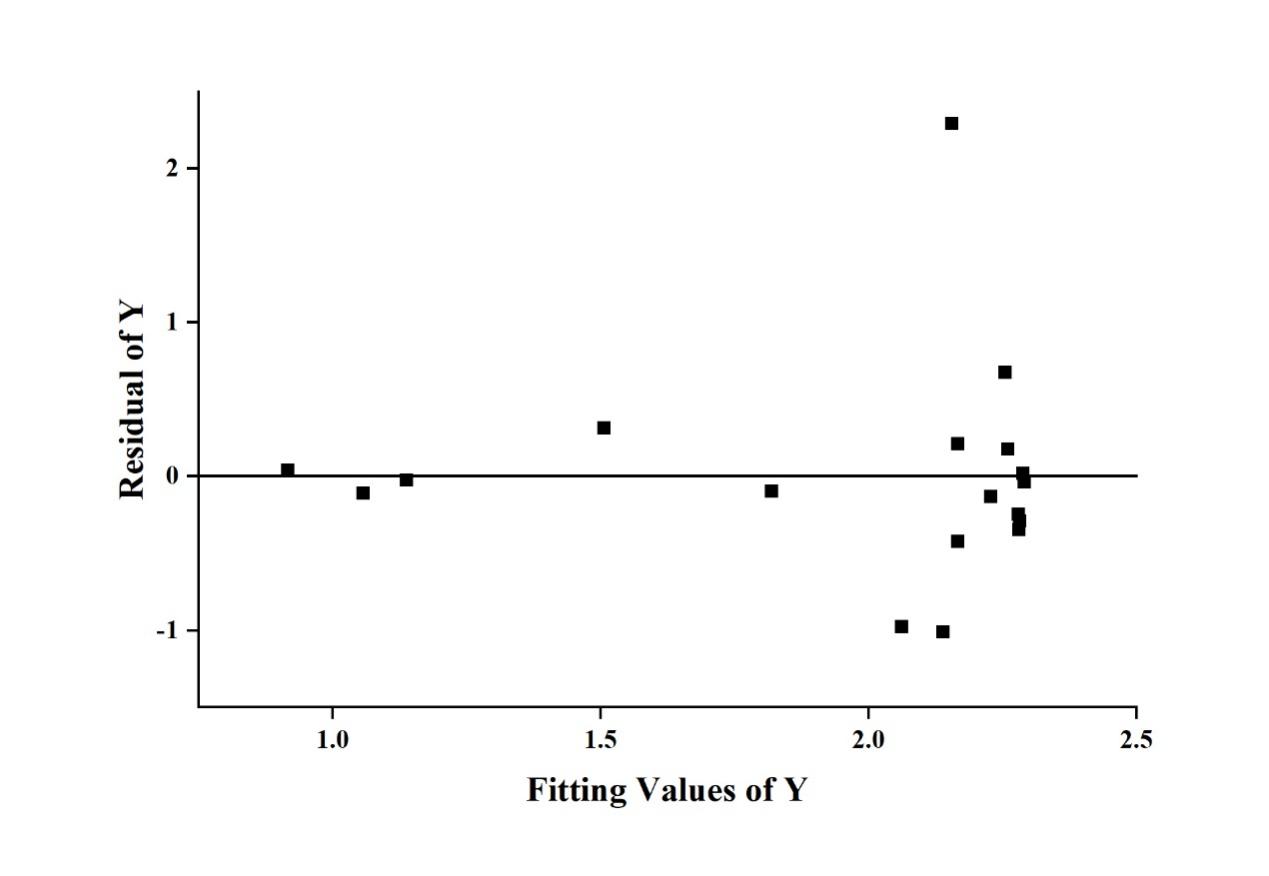
**

**Fig. S6**

**Table S1.** Comparison of fitting degree between unary function, sine function and logarithmic function by Akaike Information Criterion (AIC) and Bayesian Information Criterion (BIC). The lower values of AIC and BIC, the better the fitting degree is.

|  | Unary Function | Sine Function | Logarithmic Function |
| --- | --- | --- | --- |
| Akaike Information Criterion (AIC) | **-4.42** | 4.86 | 1.15 |
| Bayesian Information Criterion (BIC) | **-3.46** | 4.31 | 0.61 |
